# The Hard Problem of Cooperation

**DOI:** 10.1371/journal.pone.0040325

**Published:** 2012-07-09

**Authors:** Kimmo Eriksson, Pontus Strimling

**Affiliations:** 1 Centre for the Study of Cultural Evolution, Stockholm University, Stockholm, Sweden; 2 School of Education, Communication and Culture, Mälardalen University, Västerås, Sweden; Queen Mary, University of London, United Kingdom

## Abstract

Based on individual variation in cooperative inclinations, we define the “hard problem of cooperation” as that of achieving high levels of cooperation in a group of non-cooperative types. Can the hard problem be solved by institutions with monitoring and sanctions? In a laboratory experiment we find that the answer is affirmative if the institution is imposed on the group but negative if development of the institution is left to the group to vote on. In the experiment, participants were divided into groups of either cooperative types or non-cooperative types depending on their behavior in a public goods game. In these homogeneous groups they repeatedly played a public goods game regulated by an institution that incorporated several of the key properties identified by Ostrom: operational rules, monitoring, rewards, punishments, and (in one condition) change of rules. When change of rules was not possible and punishments were set to be high, groups of both types generally abided by operational rules demanding high contributions to the common good, and thereby achieved high levels of payoffs. Under less severe rules, both types of groups did worse but non-cooperative types did worst. Thus, non-cooperative groups profited the most from being governed by an institution demanding high contributions and employing high punishments. Nevertheless, in a condition where change of rules through voting was made possible, development of the institution in this direction was more often voted down in groups of non-cooperative types. We discuss the relevance of the hard problem and fit our results into a bigger picture of institutional and individual determinants of cooperative behavior.

## Introduction

Social dilemmas, or collective action problems, are situations where there is a conflict between individual and group interests so that if the individuals try to maximize their own payoff the whole group ends up with less than if they had acted in an other-regarding way [1]. This tension between rational choice and successful cooperation is the topic of a vast literature spread over all disciplines of social science.

Our point of departure is a robust finding from social psychology and experimental economics: Everyone does not behave like *Homo economicus*, the self-interested profit-maximizer. Some individuals free-ride all along but others are more cooperative-minded, although most of them eventually give up their attempts at maintaining cooperation as these attempts are exploited by the free-riders [2]–[4]. Individual variation in inclinations to cooperate is consistent with similar findings of heterogeneity in related constructs like “social value orientation” [5], [6] and “social preferences” [7].

Several experiments have shown that if cooperative-minded people get to play with other like-minded individuals in small groups they can achieve stable cooperation in repeated social dilemmas [8]–[10]. However, when members of experimental groups are not cherry-picked for cooperativeness the typical outcome is that cooperation gradually declines [11]. Thus, we cannot trust social dilemmas to be solved automatically through a universal human taste for cooperation. These results instead point to the group’s composition of “types” as a factor that determines how difficult it will be to solve the social dilemma. When all group members are of a cooperative type it is easy to achieve cooperation; the worst case is when all group members are of a non-cooperative type. We shall refer to the latter situation as *the hard problem of cooperation*. Social dilemmas in arbitrary groups then lie somewhere along the dimension between the easy problem and the hard problem.

To understand the hard problem of cooperation we must understand the nature of individual variation in cooperativeness. In the literature on social dilemmas it is often pointed out that non-cooperation is the rational choice, suggesting that people who tend to choose a non-cooperative strategy in a social dilemma are more like *Homo economicus* than those who tend to choose a cooperative strategy. One of the main points we want to make in this paper is that it is not necessarily true that non-cooperative types are more rational. As we shall discuss later there are many factors that interact in shaping individual behavior, including factors that may change rapidly over time, such as experience and beliefs, as well as more stable preferences and heuristics. Such preferences and heuristics may create both cooperative and non-cooperative behavioral patterns. For instance, when encountering a possibility to make voluntary contributions to a public good, an individual may think “This looks like the kind of situation where I typically do not contribute much” and behave accordingly. To the extent that people do not directly optimize profit but instead follow such habits, the “hard problem” may be even harder than the traditional problem of rational actors in a social dilemma.

As documented by Douglass North and Elinor Ostrom, a great range of institutions have been developed to govern behavior in a great range of social dilemmas and related situations, and these institutional solutions vary enormously in their success [4], [12]. There are several theoretical reasons why it may be difficult for rational actors to achieve a functional institution, even if they know how such an institution ought to be designed. For instance, creation of the institution may itself be a collective action problem [13]; actors may have conflicting preferences for institutions [14], and institutions may need to be trusted to become functional but must prove to be functional before they become trusted [15]. However, in the laboratory it is easy to set up a situation where an institution can be developed without any of these obstacles. We shall present such an experiment to demonstrate that even when a harsher and more demanding institution would be profitable to each individual, groups of non-cooperative types typically do not adopt it. This is remarkable given that members of non-cooperative groups are those that would benefit most from the implementation of such an institution. As we shall develop further below, the reason we predict this remarkable hardness of the “hard problem” is that non-cooperative types, because they are inclined toward low levels of cooperation, may be reluctant to support institutions that punish a behavior they often engage in (whereas *Homo economicus* would happily support such an institution as long as it leads to higher payoffs).

This paper extends the literature on institutions and their evolution by incorporating the personality dimension. In personality psychology, our approach would be labeled “interactionist” [16].

Following Douglass North and Elinor Ostrom we shall conceive of institutions as collectively shared rules about behavior that changes the incentive structure. Another common perspective in theoretical economics views institutions as equilibria in a game with multiple equilibria [17]. In our conception the institution is instead part of defining the game, which is in line with how the term is used in experimental economics. In the context of common-pool resource problems, Ostrom’s precise definition of *institutions* are “the sets of working rules that are used to determine who is eligible to make decisions in some arena, what actions are allowed or constrained, what aggregation rules will be used, what procedures must be followed, what information must or must not be provided, and what payoffs will be assigned to individuals dependent on their action” [18]. Such an institution may curb free-riding and promote cooperation by changing the strategic situation.

Extensive field studies indicate that successful solutions to real-life collective action problems do not appear out of nowhere but tend to be based on the evolution of well-designed institutions [12], [18]–[20](Note that the term “evolution” is not used in its biological sense but refers to the open-ended cultural process whereby human behavior, agreements and procedures are created and changed.) There is also an experimental literature on institutions, addressing issues of which institutions are most efficient and best preferred [26]–[30]. However, institutions that are used in laboratory experiments tend to be extremely simple compared to institutions found in the field. We shall return to this point later.

North argued that the belief systems of societies determine how institutional incentives are perceived and how institutions evolve: The belief system filters the information that actors get from current experiences in ways that induce choices that lead to institutional change, which in turn may affect beliefs [12]. Rothstein elaborated on this theme and pointed to the particular importance of beliefs about general trustworthiness and trust. Such beliefs are not given by human nature but are part of path-dependent dynamics without which one cannot “explain what is most interesting, namely the enormous variation in groups or societies ability to find solution to social dilemmas” [15]. What we argue here is that also the personality dimension will play a part in these dynamics. As we shall demonstrate, institutions with sufficiently strong and credible sanctions against free-riding can eliminate the behavioral differences between cooperative and non-cooperative types, but the desirability of such an institution may be perceived differently by different types.

Our demonstration will be a laboratory experiment where we use a standard public goods game to which is added an institutional structure that represents a stylized version of the institutions that Ostrom describe in the field [18]. We shall call an institution “strong” if the shared rules involve high expectations on cooperative behavior and sufficiently severe punishments for not meeting these high expectations so that if you are caught free-riding you will earn less than if you meet expectations on cooperation. We shall use the terms “non-cooperative types” and “cooperative types” for individuals who, when faced with a social dilemma like a public goods game, are inclined to contribute relatively little or relatively much, respectively. Following some previous studies we shall form groups of like-minded participants, such that non-cooperative types and cooperative types are in different groups [9], [10], [31]. By letting these groups play the public goods game we implement both the “hard problem” of cooperation and the “easy” version. On the one hand, we wish to demonstrate that the hard problem can indeed be solved through a sufficiently strong institution. We expect non-cooperative types to adapt their behavior to the dramatically changed incentive structure created by the institution and thereby attain a cooperative outcome. This is consistent with previous research on how low contributors adapt to the threat of punishment [31]. On the other hand, by letting the strength of the institution endogenously and gradually evolve through voting within the group, we wish to demonstrate that the hard problem is indeed hard. There is no cost involved in voting for a stronger institution, so if stronger institutions give higher payoffs then profit-maximizing actors ought to vote for them. There is nonetheless good reason to believe that non-cooperative types will not take this profit-maximizing view, because it conflicts with the natural tendency to avoid making one’s own inclinations deviant and punishable. To give two real-life examples, a study of tax evasion in Australia show that tax evaders tend to support lower, not higher, taxes [32], and another study found those less willing to serve in a war to be less in favor of punishing resistance to drafting [33]. The experimental literature on social dilemmas offers some more direct evidence. Ertan, Page and Putterman [34] studied a public goods game in which participants repeatedly voted on whether to make it possible for individuals to punish certain other individuals, and found a tendency among participants to “vote according to their type” in the sense that lower-than-average contributors in the past were less likely to vote for enabling punishing of lower-than-average contributors in the future.

We shall now explain our model and present the experimental set up and our specific predictions.

### A Model of Evolving Institutions for Collective Action

Laboratory experiments on social dilemmas usually use either a public goods game or a common-pool resource problem [11], [35]. Many experiments extend the range of individuals’ actions to include sanctions of other individuals in the form of punishments or rewards [31], [36]–[40]. There are also a few studies where participants choose whether or not they prefer the game to include the possibility for individuals to take sanctioning actions against others [34], [41]. Here we wish to make an important terminological point: The existence of individual sanctioning possibilities in the game is not in itself an institution in the Ostromian sense that we use in this paper; there must also exist some shared rules that guide sanctions.

It is well-known from experiments on social dilemmas that communication between participants facilitates cooperation [42]. An obvious function of communication is to establish shared rules. In real life, some degree of communication is typically possible and thus we ought to expect that in situations where cooperation can occur there will typically emerge shared notions about what behavior to expect and what behavior is acceptable. These shared notions include what sanctions to expect and what sanctions are acceptable [43]–[45].

At the core of Elinor Ostrom’s theory of institutions lies her identification of a number of key design features of successful, durable institutions [18]. In a most condensed form, those design features that do not involve external factors (like conditions of properties, relationships to higher governments, etc.) can be summarized as follows:

- **Operational rules.** There exist collectively known expectations on some behavior in the underlying game.
- **Monitoring.** At a relatively low cost to themselves, agents can monitor whether another agent complies with the rules.
- **Rewards.** The group rewards agents who find cheaters.
- **Punishments.** The group determines punishment of agents who are found out as cheaters.
- **Change of rules.** All agents can participate in modifying the rules.

In order to see how these feature can be implemented in the laboratory, we shall briefly discuss two experimental designs in the literature. First, Kroll, Cherry and Shogren [29] let participants vote on how much one ought to contribute in a public goods game where everyone could see how much others had contributed, and each participant could, at a cost, choose to punish others, typically cheaters. In terms of the key features, operational rules existed and could be changed, monitoring was cost-free and consequently there could be no rewards to monitorers, and punishments were not a collective concern but at the discretion of volunteering individuals. Second, Casari and Plott [26] studied a common-pool resource problem where the participants could monitor others for a cost, and if a high extractor was detected then a punishment was automatically handed out and the monitorer received a reward, but participants could not change the rules. By using features from both these designs, the model we develop below incorporates all the design features listed above.

In the following description of our model, numbers in parenthesis following a parameter is the parameter value used in our experiment. To begin with, we take the underlying collective action problem to be a public goods, PG, game where some number 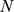 (4) of agents each obtain an endowment of 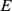 (10) units each. Each agent decides how much of her endowment to contribute to the common pot and keeps the rest. After these decisions have been made, the common pot grows by a multiplicative factor 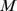 (2), with 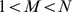. The common pot is then distributed equally to all agents. This game, which we shall refer to as the *unregulated PG game* is a collective action problem because, whereas the social optimum is achieved when all agents contribute their entire endowments to the common pot, each individual agent is better off by not contributing.

We now introduce an Ostromian institution to regulate behavior in this collective action problem.

#### Operational rule

The institution stipulates what is the smallest acceptable level of contribution to the common pot. Let 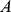 (units) denote this acceptability threshold.

#### Monitoring

Every agent can monitor at the cost of 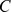 (1) units. If she chooses to monitor, then another agent is randomly drawn and is checked for rule compliance.

#### Reward

If someone who has contributed less than the acceptable level of 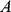 units (henceforth a “cheater”) is monitored, the successful monitorer obtains a reward of 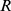 (3) units. This reward is funded by the common pot after it has been multiplied, so rewards redistribute resources to successful monitorers. If the common pot is not sufficient to cover outstanding rewards, the necessary units are taken directly from the endowments of group members. (In the experiment below this case only occurred once and the deduction was minimal, one unit.).

#### Punishment

An agent who is found out as a cheater (through someone else’s monitoring) is automatically punished by a fine of 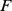 units. These units disappear, so for the rest of the group the punishment is associated neither with a direct cost nor a direct benefit. (Thus, following Casari and Plott [26], no individual bears any cost for the punishment of someone else. We may think of the collective as ascribed with the power to sanction rule infractions, which is consistent with how some communities have self-organized [18].).

We shall refer to this scenario as the *regulated PG game*. Viewed as one-shot games, the unregulated PG game has a unique pure Nash equilibrium with zero contributions, whereas the regulated PG game typically has a mixed equilibrium where players either try to cheat by contributing zero or contribute at the lowest acceptable level of 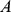 units. However, in our experiment the games will be repeated a number of times within the same group, so that group level differences can be studied over time. The institution may be either fixed or evolving during this time. In the latter case, agents are given opportunities to change the institution after every two rounds of regulated PG game.

#### Change of rules

Agents get to vote on whether to change the value of a given institutional parameter. There are three options: first, raising the parameter value by one unit; second, keeping it at its current level; third, lowering it by one unit. If either raising or lowering the parameter value is strictly more popular than any of the other two options, the value is changed accordingly.

In our experiment we shall investigate the effect of manipulating the values of the acceptance threshold 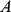 and the size of the fine 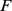 as well as the effect of allowing the values of these parameters to evolve through voting. The reward for successful monitoring is kept fixed at 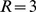 units.

### Predictions and Experimental Design

As outlined above, our research aimed at determining the importance of differences in “inclinations” or “type” with respect to behavior in an institutionally regulated collective action problem. To do this we must first sort participants into types. In our experiment we achieved this by letting participants play an initial stage of the unregulated PG game. We divided participants into groups depending on how much they contributed in the initial game. Consistent with the body of previous research on the PG game, we expected participants to exhibit substantial individual variation in their initial contributions to the common pot, so that a *low* group of individuals inclined to make very low contributions could be distinguished from a *high* group of individuals inclined to make substantially higher contributions. We think of the low and high groups as being made up of non-cooperative and cooperative types, respectively. With respect to these types, we made the following predictions concerning collective action problem behavior and institutions.

#### Prediction 1

With *no* institution, i.e., in an unregulated PG game, types will play a large role: low groups will achieve much less cooperation than high groups. This prediction is in line with prior research findings [9].

#### Prediction 2

Under a fixed *weak* institution, in the sense that quite low contribution levels are acceptable (i.e., low 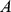 value), types will still play a large role. The reason is that cooperative types are not constrained from following their inclinations to contribute above the acceptance threshold.

#### Prediction 3

Under a fixed *strong* institution, in the sense that only very high levels of contributions are acceptable *and* cheaters risk sufficiently high fines (i.e., high values of 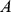 and 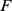), the incentive structure makes it rational to cooperate at high levels if you believe you will be monitored. Thus a strong institution ought to eliminate the behavioral differences between types so that all groups achieve high cooperation levels. Because cooperative types were expected to cooperate rather well even under the weak institution (Prediction 2), these two predictions together say that it is the non-cooperative types who will gain most from playing under a strong rather than a weak institution.

#### Prediction 4

If a weak institution is not fixed but is free to *evolve* through voting on parameter changes, then low groups will be worse than high groups at developing a strong institution. Consequently, there will remain a difference in cooperation levels between low and high groups.

The rationale of the last prediction was discussed earlier, in terms of non-cooperative types being likely to have a tendency to avoid making their own inclinations deviant and punishable. Another way of expressing this is that we expect people to be myopic when evaluating a potential institutional change, so that they do not sufficiently take into account how they could benefit if everyone’s behavior (including their own) adapted to the institutional change. (Consistent with theoretical and experimental results on voting behavior [46] we expect people to vote according to their preferences despite the uncertainty of whether their vote will matter.).

Together these predictions tell a fundamental story about the double-edged relation between institutions and types: On the one hand, strong institutions have the power to eliminate behavioral differences between types, such that groups that would otherwise free-ride benefit the most. On the other hand, types will affect the evolution of institutions, such that the groups that would benefit the most from developing a strong institution are the least likely to succeed in doing it.

The second phase of the experiment was designed to test the above predictions. Within high and low groups, participants played the PG game in a sequence of conditions.

- **Stage 1 (** ***evolving institution*** **).** Sixteen rounds of the regulated PG game with voting after every second round; players vote within their group on how to change the acceptance threshold and the fine level (separate votes). The institution starts weak, i.e., at parameter values 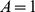 and 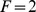. At the end of the game, the parameter values can reach 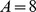 and 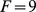 if voting consistently results in increased parameter values.
- **Stage 2 (** ***weak/strong institutions*** **).** A total of eight rounds of the regulated PG game with fixed institutions in a sequence of two conditions (randomly ordered). The weak institution condition has four rounds with fixed parameter values 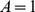 and 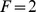; the strong institution condition has four rounds with 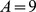 and 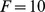.
- **Stage 3 (** ***no institution*** **).** Four rounds of the unregulated PG game.

It is informative to look at the one-shot equilibria of these games, which can be compared with final round behavior in the experiment. In the unregulated PG game, the one-shot equilibrium is, of course, for everyone to make zero contributions, resulting in a payoff of 10 units per round. In the Materials and Methods section, a one-shot equilibrium analysis of the regulated PG game is conducted. According to this analysis, the expected payoffs per round in equilibrium in the weak and strong institution conditions are 10.2 units and 12.7 units, respectively. In the evolving institution condition, the same analysis says that profit-maximizing agents who expect equilibrium outcomes will consistently vote for higher parameter values and thereby achieve an expected equilibrium payoff of 12.4 units in the final round.

## Results

### Effects of Institution and Group Type on Cooperative Behavior

The payoff of a participant in any given round is the net amount of units made in the game when all decisions and sanctions are taken into account. To assess the level of cooperation achieved in various groups and conditions, we looked both at contributions and payoffs. To assess predicted differences between low and high groups we used one-tailed paired samples t-tests (Wilcoxon signed rank tests give similar results) on the group data from the final periods of each condition (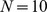 pairs of groups). Table 1 report, for low and high groups, the mean payoffs, contributions and monitoring. The results are group totals divided by four, the number of group members, representing the average individual behavior. We shall discuss these results for each condition in turn. Analysis of the average over all periods give similar results that are summed up in Table 2 and Table 3.

**Table 1:** Mean (SD) payoffs, contributions and monitoring (group totals divided by the group size, i.e., 4) in the final period of each condition.

	Payoff		Contribution		Monitoring	
Institution	low groups	high groups	low groups	high groups	low groups	high groups
Evolving	13.28 (2.88)	16.80 (1.67)	4.55 (1.78)	6.88 (1.20)	0.62 (0.27)	0.40 (0.21)
Weak	11.45 (1.12)	13.58 (2.25)	1.90 (1.09)	3.88 (2.05)	0.40 (0.27)	0.20 (0.20)
Strong	16.92 (2.15)	16.95 (2.04)	7.65 (1.54)	7.98 (1.54)	0.48 (0.18)	0.52 (0.32)
No	11.95 (1.14)	15.52 (2.17)	1.95 (1.14)	5.52 (2.17)		

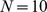
 groups of each type.

**Table 2:** Mean (SD) payoffs, contributions and monitoring (group totals divided by the group size, i.e., 4) averaged over all periods in each condition.

	Payoff		Contribution		Monitoring	
Institution	low groups	high groups	low groups	high groups	low groups	high groups
Evolving	12.85 (0.77)	15.78 (1.12)	3.79 (0.51)	6.36 (0.90)	0.55 (0.12)	0.43 (0.13)
Weak	11.55 (1.13)	13.77 (2.06)	2.05 (1.06)	4.06 (1.88)	0.41 (0.24)	0.24 (0.18)
Strong	16.64 (1.64)	16.60 (1.60)	7.78 (1.03)	7.94 (0.90)	0.52 (0.21)	0.59 (0.18)
No	12.09 (0.87)	15.12 (1.55)	2.09 (0.87)	5.12 (1.55)		

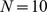
 groups of each type.

**Table 3:** Differences between the high and low group in payoff, contribution and monitoring averaged over all periods in each condition (group totals divided by the group size, i.e., 4).

	Payoff		Contributions		Monitoring	
Condition	difference	t value (p value)	difference	t value (p value)	difference	t value (p value)
Evolving	2.93	5.31 (.000)	2.58	6.67 (.000)	−0.13	−1.89 (.046)
Weak	2.22	2.49 (.018)	2.02	2.51 (.016)	−0.18	−1.83 (.051)
Strong	−0.04	−0.05 (.483)	0.16	0.34 (.372)	0.08	0.77 (.232)
No	3.03	5.11 (.001)	3.03	5.11 (.001)		

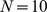
 groups of each type.

In the *evolving institution* condition, high groups achieved higher levels of cooperation than low groups, with a 2.3 units difference in contributions, 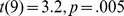, and a 2.8 units difference in payoffs, 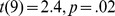.

In the *weak institution* condition, the corresponding difference in contributions was 2.0 units, 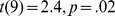, and the difference in payoff was 2.1 units 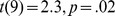. However, in the *strong institution* condition, differences virtually disappeared and were statistically insignificant (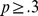). The effect of institution strength (strong vs. weak) was larger for low groups: compared to high groups, they achieved a 1.6 units higher increase of contributions, 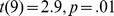; they also achieved a 2.1 units higher increase of payoffs, 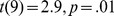. (Comparison of sessions differing in the order of the weak and strong institution conditions revealed no order effects.).

In the *no institution* condition, contributions were 3.6 units larger in high groups than in low groups, 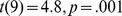. In this condition monitoring was not possible and mean payoffs per group member was therefore necessarily 10 units plus the mean contribution per group member because of the doubling of the common pot. Note that the contributions in the high groups tended to be lower under the weak institution than under no institution. This might simply be an order effect but it might also be the case that the weak institution has a normative effect, such that people contribute less than they would otherwise have done because that is what they perceive the norm to be. This is an interesting topic for future investigations.

To summarize, behavioral types played a large role, such that groups of non-cooperative types cooperated much less, not only in the absence of an institution (Prediction 1) but also under a weak institution (Prediction 2). Under a strong institution this difference between types was eliminated, and this was particularly beneficial for the groups of non-cooperative types (Prediction 3). When the groups were given the opportunity to develop the institution on their own, the difference in cooperation between types was not eliminated (Prediction 4).

### Effect of Group Type on the Outcome of Institutional Change

In Prediction 4, group differences in the evolving institution condition was expected as a consequence of a predicted difference in institutional change between low and high groups. Specifically, we expected members of low groups to be more reluctant to increase the fine level 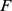. Here we analyze this part of the prediction.

**Figure 1:**
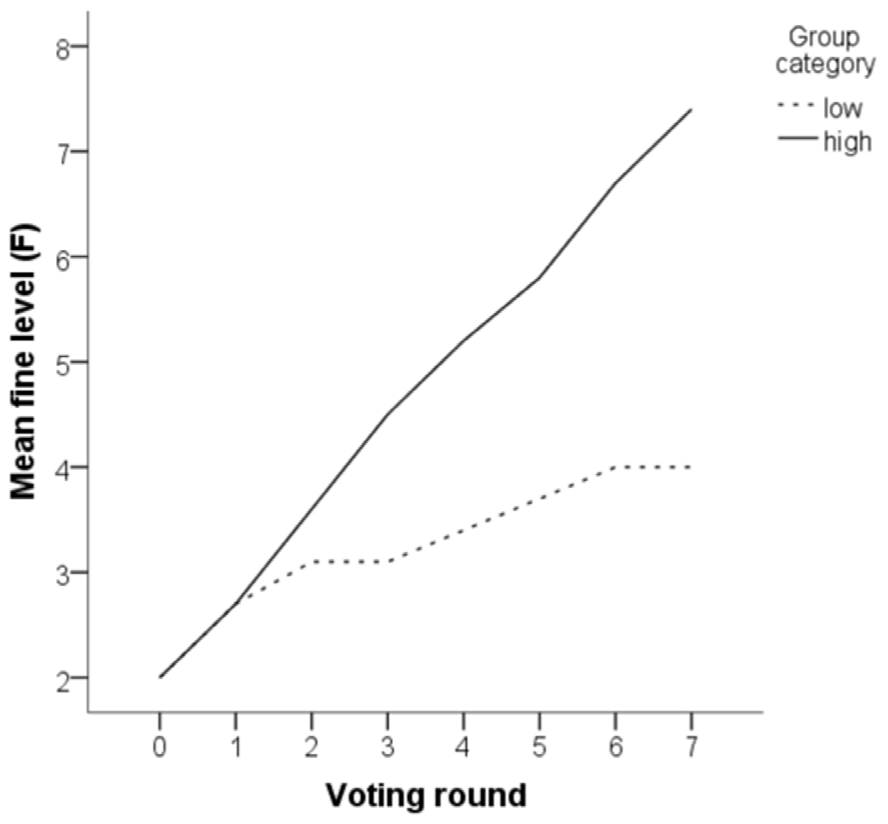
Evolution of the parameter *F* **over the seven rounds of voting in stage 1 (evolving institution).**

Figure 1 shows how the fine level 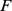 changed over time in low and high groups, illustrating a dramatic difference in the predicted direction. High groups raised the fine for free-riding at almost every occasion whereas low groups did not. At the end of the game, the fine level in high groups (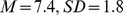) tended to be much higher than in low groups (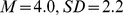), 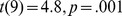. This pattern was very robust, with the high group finishing at a higher fine than the low group in nine sessions out of ten, and in the tenth session the high and low groups finished at the same level.

**Figure 2:**
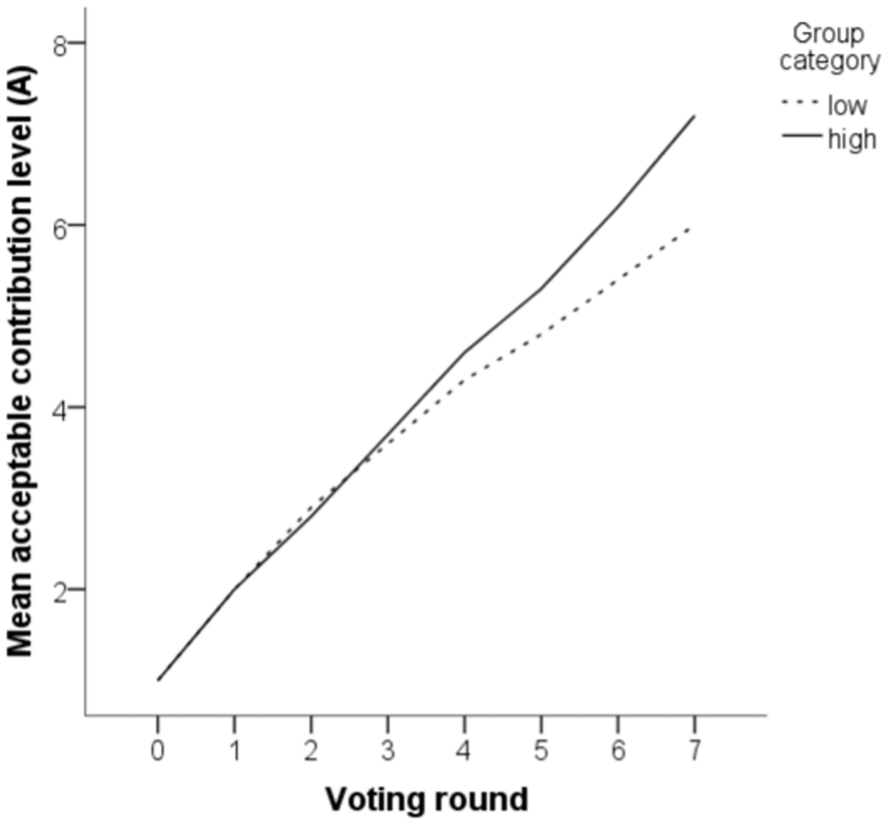
Evolution of the parameter *A* **over the seven rounds of voting in stage 1 (evolving institution).**

Group type had a small effect in the same direction also for the evolution of the acceptance threshold 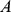 (Figure 2). At the end of the game, the acceptance threshold tended to be higher in high groups (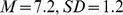) than in low groups (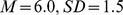), 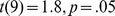. In eight sessions out of ten the acceptance threshold finished at a higher level in the high group than in the low group (perhaps parallelling the finding that tax evaders tend to support lower, not higher, taxes [32]).

### Comparison with Equilibrium Behavior

We also compared payoffs in the final period with the expected payoffs in the corresponding one-shot equilibria, which are calculated in the Materials and Methods section to be 10 units for *no institution*, 10.2 for *weak institution*, 12.7 for *strong institution*, and 12.4 for *evolving institution*. In the high groups, actual payoffs were always considerably higher than these equilibrium payoffs (each difference was at least 3.4, all 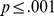). In the last three conditions (weak, strong and no institution), actual payoffs were higher than equilibrium payoffs also for low groups (each difference was at least 1.2, all 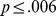). However, for low groups in the evolving institution, the difference was small (0.9) and statistically insignificant, 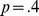. We conclude that low groups on the whole behaved more cooperatively than *Homo economicus* but that this advantage in the evolving institution condition was offset by the low groups’ poor development of the institution.

The strong institution condition showed the most remarkable difference between actual payoffs and equilibrium payoffs, with both high and low groups achieving mean payoffs at 16.9 units, two standard deviations above the equilibrium payoff (12.7). Why did both high and low groups achieve such high levels of cooperation under the strong institution? One answer is obtained through comparison of frequencies of cheating and monitoring with the equilibrium frequencies. Across all periods of this condition, the cheating frequency was much less (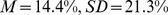) than in equilibrium (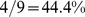), 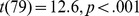, which is strategically consistent with the monitoring frequency being higher (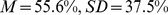) than in equilibrium (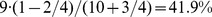), 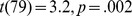. Thus, from a strategic perspective the surprising behavior is that monitoring is common despite low frequencies of cheating. A lower than equilibrium level of cheaters should lead to a lower than equilibrium level of monitoring. The reason for over-monitoring could be that some participants, in addition to taking payoffs into account, have a preference for punishment of others who cheat [31], [37], [39].

## Discussion

**Figure 3:**
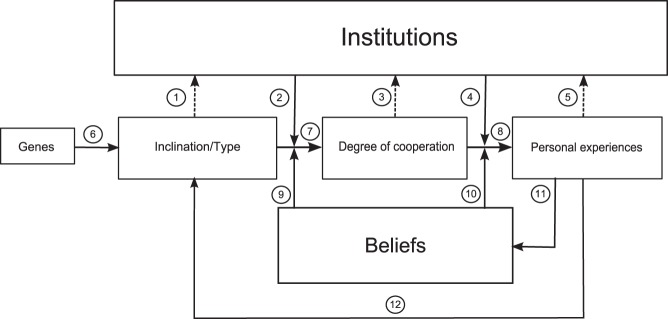
How cooperative behavior and some of its major determinants may affect each other.

Research on cooperation is conducted within several schools of thought that focus on different kinds of explanations. Moreover, experimental research on cooperation has a tendency to focus on particular factors without any specifications of how these factors fit with each other. As the interpretation of our results relies on ideas from several lines of research, we feel a need for a general framework within which we can place both previous research and our new findings. Figure 3 shows our attempt at such a general framework. The diagram models the interconnections of determinants of the degree to which an individual cooperates in a given repeated social dilemma. The lower part represents factors manifested at the individual level (genes, inclinations, behavior, experiences and beliefs) whereas the institution on top is manifested at the collective level. As discussed earlier, we here conceive of institutions as including general expectations about how to behave and what kinds of monitoring and sanctioning are legitimate; depending on the specific social dilemma and the specific group it concerns, such institutions may be more or less formalized [18], [19]. Dashed arrows represent how factors on the individual level influence the way the individual will contribute to institutional change. Horizontal arrows represent effects on the individual level; vertical arrows represent how these effects are moderated both by the institution and by the individuals’ beliefs.

In our experiment we investigated three of these arrows: how inclinations determine degree of cooperation (arrow 7), how this effect is moderated by the institution (2), and how individuals’ inclinations affect institutional change (1).

The role of beliefs in the diagram is an attempt at capturing the message of North [12] and Rothstein [15]: Beliefs are crucial to how individuals perceive and interpret experiences (10) and how they will choose to behave in a social dilemma (9), and beliefs are modified through personal experience (11).

As to the origin of individual variation, the working assumption of some evolutionary oriented researchers [47]–[51] have been that types are genetically based (6). However, new research on the heritability of cooperative behavior in specific games played in the laboratory typically finds that genetic differences explain only about twenty percent of individual variation [52], [53]. Thus, we must expect behavioral inclinations to be largely learned and flexible (12), which is consistent with other lines of evidence [4], [54]. Of particular interest for the topic of the present paper is recent cross-cultural research that has found population differences in behavioral inclinations consistent with a systematic influence of institutions [21], [55]–[57]. In our schema this effect is represented as the path from institutions to inclinations via experiences.

The three dashed arrows in Figure 3 represent that the institution is not fixed and that its destiny depends on several factors on the individual level, of which we have already discussed inclinations (1). The individual’s exhibited degree of cooperative behavior (3) is in itself part of the institution’s “descriptive norm” [45], which through people’s experiences will feed into their beliefs about how people in general behave. The last arrow (5) represents that an individual’s personal experiences of living under a certain institution may provide motives to prefer a certain change [19], [58]. Altogether, we find Figure 3 a helpful tool for thinking about how research on determinants of cooperation fits together and how these factors constitute a dynamic system with feedback loops.

We now turn to a discussion of the specific results of our study. In this paper we have experimentally studied cooperative and free-riding behavior in a well-known social dilemma regulated by an Ostromian institution. Under a weak institution, individual types mattered a great deal for the outcome; under a strong institution, individual types did not matter anymore; and under an institution that could evolve gradually through voting, differences in individual types mattered because they led to different institutional outcomes. These results demonstrate that in order to explain variation in human cooperation we must consider both institutional and individual variation.

In the introduction we coined the term “the hard problem of cooperation” for the problem of making a group of non-cooperative types achieve a high level of cooperation in a social dilemma. Our results illustrate an important and non-trivial sense in which the hard problem is hard. Under a weak institution, where expectations and punishments are low, groups of non-cooperative types achieved very low levels of cooperation and earnings. A strong institution made the same groups achieve very high levels of cooperation and earnings, showing that a properly designed institution can indeed solve the hard problem. The individual monetary gain from changing from a weak to a strong institution was much larger in these groups of non-cooperative types than in groups of cooperative types. Even so, it was the groups of non-cooperative types that did not succeed in developing the institutional strength to high levels when given the opportunity to do so. Thus, the non-cooperative types tended to not vote for increasing punishments for free-riding. This finding shows that non-cooperative types do not behave like *Homo economicus* with respect to choices of institution; raising their individual payoffs by curbing others’ free-riding tended to be a lesser concern for them than their personal aversion for such an institution. Studies of behavior in public goods games have found that there is variation not only in the dimension we have studied here (i.e., initial levels of contributions) but also in the the extent to which cooperators are :conditional” (i.e., decrease their contributions if others make low contributions). Further research might address whether differences along this dimension, which can be thought of as how easily one adapt one’s behavior to others, play a role also when adapting one’s behavior to a new institution.

Does the hard problem exist outside the laboratory? We think it is likely that instances of the hard problem are quite prevalent. The groups of “non-cooperative types” in our study constituted the lowest-contributing third among the twelve participants in each session, so these were not very special individuals. Further, the participants were recruited among students in Sweden, a country of very high trust levels and very low levels of perceived corruption [59], [60]. Above we cited evidence that behavioral inclinations are malleable and may be systematically affected by the experiences induced by institutions of different quality, which suggests that countries with less well-functioning institutions may have a higher frequency of hard problem individuals. Of particular interest in this regard is a study of Herrmann, Thüni and Gächter [21]. They had participants in fifteen countries play a repeated public goods game with and without a possibility to sanction others. Inefficient (“anti-social”) use of punishments turned out to be higher among participants in countries with poorer rule of law, and higher use of anti-social punishment correlated with lower contributions in the game with sanctions. To highlight our point, we shall analyze their data in a different way. Table 4 presents for each country how much average contributions in the public goods game increased when the sanctioning possibility was added. This increase reflects how well participants managed to use the sanctioning possibility to establish an efficient informal institution. In the same table we also present, for each country, the Corruption Perception Index (CPI) for the relevant year [59]. CPI measures perceptions of the degree of corruption as seen by business people and country analysts, and ranges between 10 (highly clean) and 0 (highly corrupt). The two columns of Table 4 are strongly correlated (Pearson’s 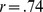, 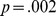), indicating that participants from less corrupt countries were more successful at establishing an efficient informal institution. CPI and rule of law are of course closely related. In a multiple regression with both CPI and rule of law as predictors, only CPI came out as a statistically significant predictor, suggesting that perceived corruption may be the more important concept to explain country differences in use of sanctions in games.

**Table 4:** Corruption Perceptions Index and average effect of peer-to-peer sanctioning on PG contributions.

Country	CPI	Contribution increase
Australia	8.6	9.2
Belarus	2.1	2.4
China	3.5	5.9
Denmark	9.4	6.2
Germany	7.8	5.3
Greece	4.6	−0.7
Oman	4.7	−0.1
Russia	2.5	2.0
Saudi-Arabia	3.4	−0.7
South Korea	5.1	6.8
Switzerland	9.0	6.9
Turkey	4.1	1.7
UK	8.7	8.1
Ukraine	2.8	0.3
USA	7.2	8.7

Rothstein [15] discusses endemic corruption in a society as a “social trap”. It seems likely that societies caught in such social traps will foster individuals whose inclinations are shaped by the poor institutions that are currently in place. Such situations would then have similarities to the hard problem, and we would then expect that also many individuals who would on the whole benefit from a less corrupt society might nonetheless refrain from supporting institutions that would enforce it. In order to understand whether a proposed measure to escape a social trap is likely to work, it seems to us that one must take into account the dynamics of the whole system sketched in Figure 3.

## Materials and Methods

### Ethics Statement

Ethics approval was obtained from the Regional Ethical Review Board in Uppsala, Sweden. Written informed consent was received from all participants.

### Participants and Procedure

The study was conducted at a laboratory for economic experiments at a Swedish university. One hundred and sixteen participants (54 women and 62 men, 24 years old on average) were recruited from a pool of volunteering college students from miscellaneous study programs to take part in one of ten experimental sessions. Nine sessions had twelve participants and one session had eight participants. On arrival, participants were immediately led to separate cubicles; at no time during the experiment did they see each other. Instructions on general behavior in the lab and specific instructions about the game to be played were presented on the computer, including a full tutorial with test questions that participants must pass before they could advance.

In the first phase, participants played an unregulated PG game for four rounds (in fixed groups). Based on their average contributions in this game, the four overall lowest and four overall highest contributors were categorized as “low” and “high”, respectively; remaining participants were categorized as “middle.” Mean contributions were 3.3 units for low participants, 8.7 units for high participants, and 6.4 for middle participants. The advantage of basing categories on the average of four rounds of play rather than just a single round is, of course, decreased within-participant random noise. The drawback is some between-participant random noise instead, as participants in the later three rounds can react to previous contributions of others. In any case, the difference in outcome between the alternative categorization schemes is not large; on average, less than one participant per session who were categorized as low would have been categorized differently if categorization had instead been based only on first round contributions or only on last round contributions. Thus, we do not expect results to strongly depend on the specific method of categorization.

For the second phase, low and high participants were informed that they would now continue to play the game in a new group. The basis for the division into groups was never mentioned. The middle group proceeded with an unrelated task. Thus, the second phase had one low and one high group for each of the ten sessions, totalling 80 participants.

At the end of the session participants were called out one by one to an adjoining room where they were debriefed, paid and dismissed. Average earnings were 126 Swedish kronor, about 20 US dollars.

### Instructions

In the first phase, the subject where given instructions that defined the rules of the public goods game.

You will be a member of a group of four people who will play a game. In this game each member receives 10 tokens in each round. You have to choose how many of these tokens to invest in a project. You get to keep the tokens that you do not invest in the project. For each token that you invest in the project, you and every other group member receives 0.5 tokens. Thus, since there will be four players, each token invested in the project is transformed into 2 tokens, which are split equally among all four players regardless of how much they have contributed. For instance, if you have invested 7 tokens, then you keep 3 tokens. Nobody except you earns something from these 3 tokens. From the amount you invested in the project, each group member will get the same payoff. You will also get a payoff from the tokens that the other group members invested in the project. If the sum total of investments in the group project is 20 tokens, then you and every other member of the group will get a payoff of 20*0.5 = 10 tokens from the project, so in total you will receive 3+10 = 13 tokens in this round. You will play this game four times with the same three people.

Participants then answered four control questions and could not move on until they had answered all questions correctly.

Remember! You start each round with 10 tokens. You get to keep what you do not invest in the project. Each token invested in the project yields 0.5 tokens to each player. Please answer the following questions. Their purpose is to make you familiar with the calculations of incomes that result from decisions about the allocation of the 10 tokens.

1. How much do you get for each token that another group member invests in the group project?
2. How much do you lose by investing a token in the project instead of keeping it?
3. If no one in your group invests anything in the project, how many tokens do you get in this round?
4. If everyone in your group invests his entire endowment (10 tokens) in the project, how many tokens do you get in this round?

Participants then played the public goods game for four rounds. After each round they received feedback on the total contribution to the common pot and own earnings. Those who were selected to participate in the second phase were told that they were now to play with a new group of people and that the game would now be governed by rules that would apply to all players and be subject to voting.

The rules state: (a) The acceptable level of contributions; anyone who contributes less than this level risks being subjected to a fine. (b) The level of the fine.

You will have the possibility to pay a token to monitor a randomly drawn player in your group. If this player has not contributed at least at the acceptable level, he or she is automatically fined.

Thus, if you contribute too little, and if anyone monitors you, then you are sanctioned. If you monitor someone who turns out to have contributed too little, you get 3 tokens. This payment for monitoring is paid out of the project pot after it has grown. The amount in the project pot will be visible throughout the game. You have ten tokens at your disposal. You choose how many tokens to invest in the project (if you contribute less than the required amount, you risk being sanctioned). You also choose whether you want to spend one token on monitoring another player. The program then randomly assigns a player to you to monitor. The program also computes how many tokens you keep. Everyone who monitors gets assigned a different player.

The current rules were always in display on the screen. Feedback after each round included: the total contribution to the common pot; whether you earned a reward; whether you were monitored and if so whether you were punished; how many players in this group contributed less than the threshold and how many of them were punished; how many chose to monitor; your payoff and the average payoff in the group. After every second round, players were given the opportunity to vote on the rules:

Do you think the amount required to invest in the project to avoid possible sanctions should be (a) increased by one token; (b) decreased by one token; (c) left as it is?

Do you think the sanction should be (a) increased by one token; (b) decreased by one token; (c) left as it is?

After sixteen rounds, participants were told how much they had earned in total in this part of the experiment and that a new part were about to commence where rules would be fixed (i.e., not subject to voting). They were randomly assigned to start with either the weak or the strong institution. After four rounds participants were again told how much they had earned in total in that part of the experiment and that the rules would now be changed (from weak to strong or vice versa). After four rounds with these new rules, participants were told that they would play four rounds without any rules in place (no institution).

### Formal Analysis of the Game

We shall here make a formal game-theoretic analysis of the one-shot regulated PG-game in terms of the model parameters 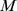, 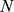, 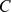, 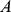, 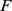, and 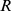.

Let 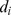 denote agent 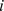
’s contribution to the common pot, and let 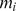 be an indicator variable of whether or not agent 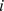 invested 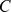 units in monitoring another’s contribution. A pure strategy in this game is a pair 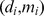 of nonnegative integers such that 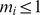 and 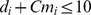. From such a strategy a cheater indicator variable 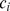 can be defined by 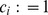 if 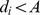 and 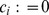 if 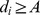.

Payoff to agent 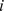 comes from units kept by the agent and from the division of the common pot after multiplication and deduction of rewards, plus any fines and rewards to the agent. Under the standard assumption that 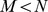 in a public goods game, only two contribution levels are possible in equilibrium: 0 and 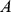. The reason is that all contributions less than 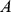 makes the agent a cheater and puts him at the same risk of fines, so all contributions strictly between 0 and 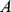 are dominated by the contribution of exactly 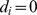 units. Similarly, all contributions greater than or equal to 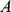 makes the agent a non-cheater and removes the risk of fines, so all contributions strictly greater than 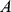 are dominated by the contribution of exactly 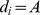 units. In the following analysis only these two strategies are considered, so that “cheating” refers to contributing 0 and “not cheating” refers to contributing 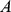 units. The payoff to agent 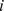 can then be expressed as follows:
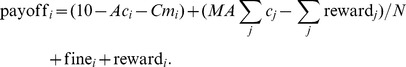
(1)


Let 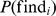 and 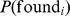 denote the probabilities of agent 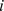 finding a cheater (if he monitors) and being found out as a cheater (if he is one), respectively. Then expected fines and rewards to agent 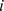 are given by
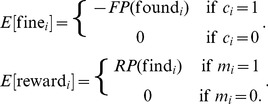
(2)


The expected profit from cheating compared to not cheating can now be expressed as
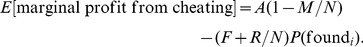
(3)


Similarly, the expected profit from monitoring compared to not monitoring is
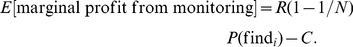
(4)


Because monitoring reveals the behavior of a randomly selected other agent, the probability of finding a cheater equals the frequency of cheating among the other agents:
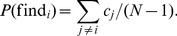
(5)


Because these random selections are made such that every agent has exactly one randomly selected designated potential monitorer, the probability of being found equals the frequency of monitoring among the other agents:
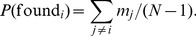
(6)


Equilibrium behavior of agent 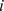 can now be described by the following two conditions:
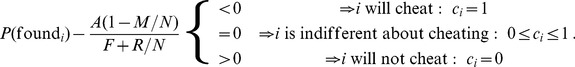
(7)

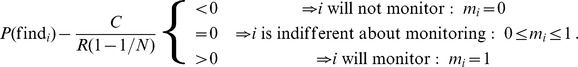



#### Proposition 1

If 
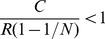
 and 
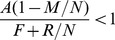
 *then the game has a unique symmetric mixed equilibrium where every agent cheats with probability 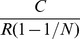 and monitors with probability 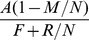. The expected payoff in this equilibrium is*

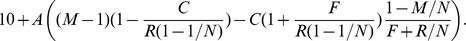



#### Proof

Suppose every agent cheats with probability 
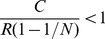
 and monitors with probability 
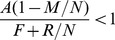
. It follows from equations (5) and (6) that the probabilities of finding and being found as a cheater is 
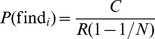
 and 
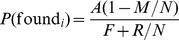
, respectively. Equations (7) and (??) imply that this is indeed a mixed equilibrium.

To see that no asymmetric equilibrium is possible, assume (in order to obtain a contradiction) that there exists an equilibrium in which agent 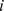 monitors more than average. It follows that 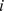 is monitored less than average, hence will cheat more than average, hence will have less than average chance to find a cheater, hence will monitor less than average, contradicting the original assumption.

The expected payoff in the symmetric equilibrium is obtained from equation (1) by plugging in 
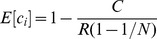
, 
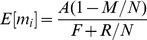
, 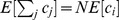 and 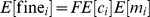. Rewards are just redistributions among the players so in the symmetric equilibrium they cancel out.

In our experiments, we have fixed parameter values 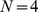, 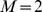, 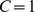 and 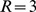. The equilibrium conditions in the theorem then simplifies to 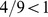, which always holds, and 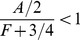, which holds whenever the fine 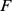 is not less than 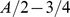. The expected payoff in equilibrium simplifies to 
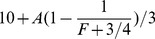
. This equilibrium payoff is approximately 10.2 in the “weak institution” condition (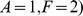, and 12.7 in the “strong institution” condition (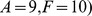. In the allowed range of parameter values (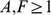) the equilibrium payoff 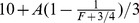 is increasing in both 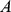 and 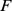, so in the “evolving institution” condition rational agents who expect the group to exhibit one-shot equilibrium behavior would vote for increasing both 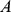 and 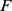 at each voting opportunity (in the process staying in the part of parameter space where the equilibrium conditions hold) and thereby in the final round reach parameter values 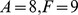, for which the equilibrium payoff is approximately 12.4.
